# Architected Metal Selenides via Sequential Cation and Anion Exchange on Self-Organizing Nanocomposites

**DOI:** 10.1021/acs.chemmater.2c03525

**Published:** 2023-03-08

**Authors:** Arno van der Weijden, Anne-Sophie Léonard, Willem L. Noorduin

**Affiliations:** † AMOLF, Science Park 104, Amsterdam 1098 XG, The Netherlands; ‡ Van ‘t Hoff Institute for Molecular Sciences, University of Amsterdam, Science Park 904, Amsterdam 1090 GD, The Netherlands

## Abstract

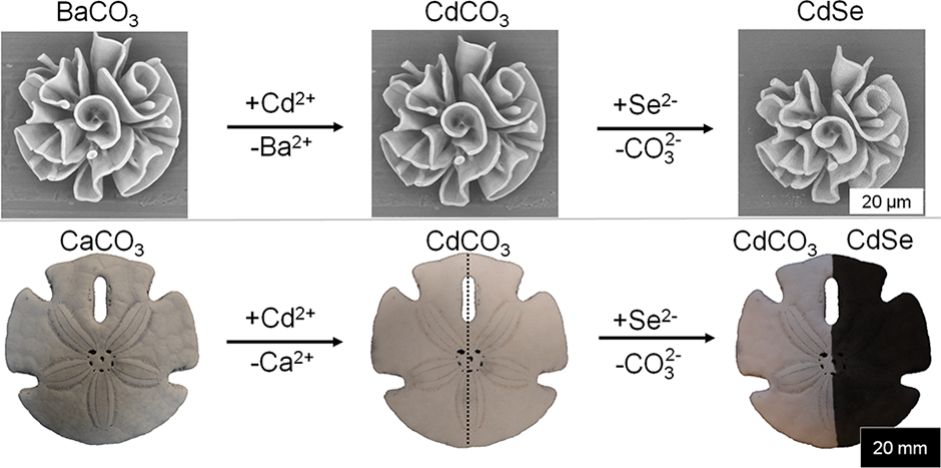

Shape-preserving conversion reactions have the potential to unlock new routes for self-organization of complex three-dimensional (3D) nanomaterials with advanced functionalities. Specifically, developing such conversion routes toward shape-controlled metal selenides is of interest due to their photocatalytic properties and because these metal selenides can undergo further conversion reactions toward a wide range of other functional chemical compositions. Here, we present a strategy toward metal selenides with controllable 3D architectures using a two-step self-organization/conversion approach. First, we steer the coprecipitation of barium carbonate nanocrystals and silica into nanocomposites with controllable 3D shapes. Second, using a sequential exchange of cations and anions, we completely convert the chemical composition of the nanocrystals into cadmium selenide (CdSe) while preserving the initial shape of the nanocomposites. These architected CdSe structures can undergo further conversion reactions toward other metal selenides, which we demonstrate by developing a shape-preserving cation exchange toward silver selenide. Moreover, our conversion strategy can readily be extended to convert calcium carbonate biominerals into metal selenide semiconductors. Hence, the here-presented self-assembly/conversion strategy opens exciting possibilities toward customizable metal selenides with complex user-defined 3D shapes.

## Introduction

**Figure 1:**
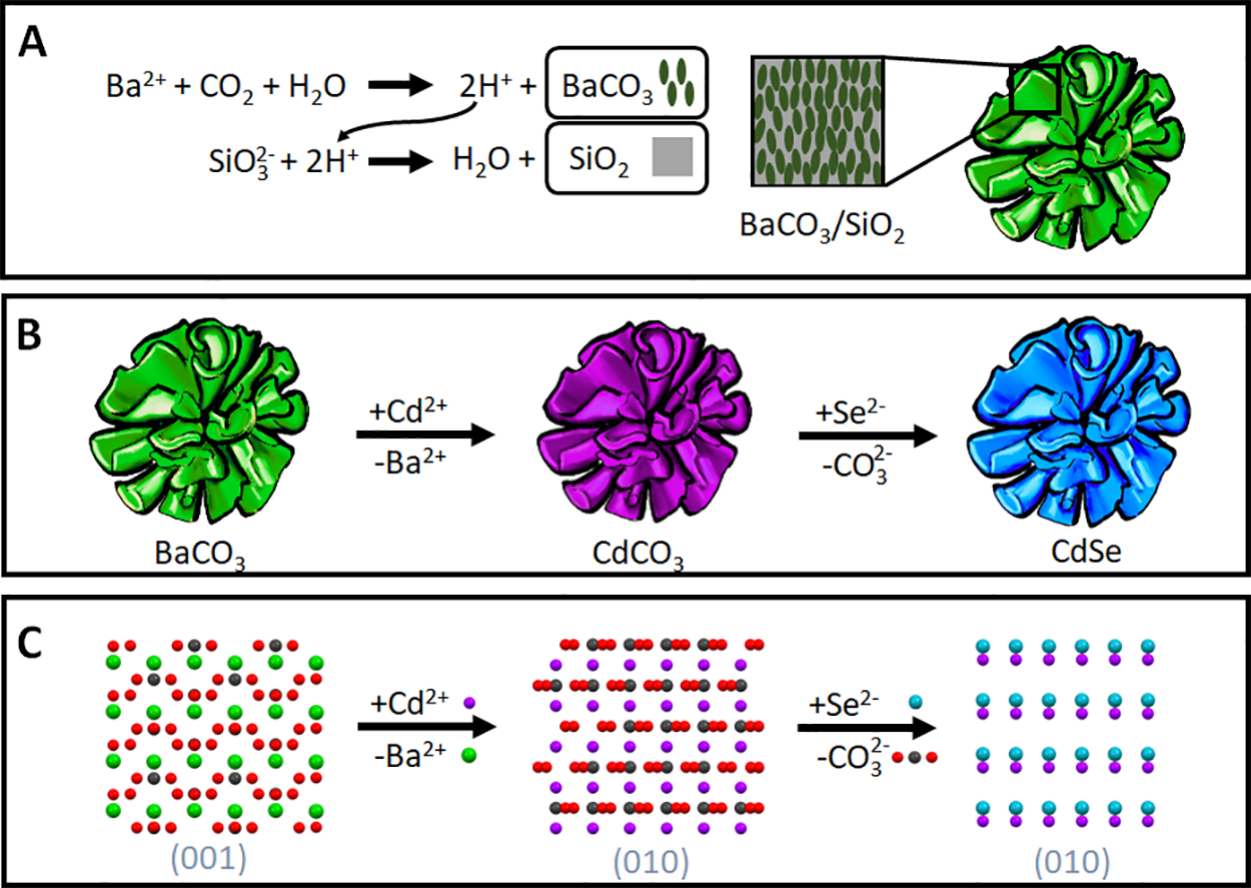
Two-step self-organization/conversion approach of architected cadmium selenide nanocomposites. (A) Acid-regulated coprecipitation of barium carbonate nanocrystals and amorphous silica into coral-shaped BaCO_3_/SiO_2_ nanocomposites. (B) Sequential ion-exchange scheme for the conversion of BaCO_3_ into CdSe via CdCO_3_. (C) Comparison of the crystal lattices showing the similarities between orthorhombic BaCO_3_, trigonal CdCO_3_, and hexagonal CdSe for facilitating the cation exchange of Ba^2+^ (green) for Cd^2+^ (purple) and anion exchange of CO_3_^2–^ (carbon (black), oxygen (red)) to Se^2–^ (blue).

The extraordinary complexity of biomineralized architectures demonstrates the possibilities for organizing a limited number of simple minerals into a wide diversity of highly refined, multifunctional, three-dimensional (3D) shapes.^1−3^ Inspired by biomineralization processes, many synthetic self-organization strategies have been developed to produce artificial complexly shaped 3D architectures.^1,4−21^ Already, a large diversity of intricately shaped 3D forms can be formed during the bioinspired coprecipitation of metal carbonate nanocrystals (MCO_3_, with M = Ba^2+^, Sr^2+^, or Ca^2+^) and amorphous silica (SiO_2_) (Figure 1A).^9,17,19^ These bioinspired nanocomposites self-organize into highly complex, yet controllable, 3D shapes such as vases, stems, helices, and coral-like forms that can be further sculpted and patterned by modulating the global reaction conditions. In addition, local control over CO_2_ concentrations using photodecarboxylation enables the steering of the self-organization process to yield metal carbonate silica nanocomposites according to exact user-defined light patterns.^22^

The resulting nanocomposites can be further chemically modified.^23−25^ Specifically, inspired by ion-exchange reactions on nanocrystals,^7,26−36^ geochemical transformations,^33^ and fossilization processes,^37^ the chemical composition of the metal carbonate nanocrystals can be completely modified using sequential ion-exchange reactions. Already, a wide range of chemical compositions have been achieved toward functional compositions such as metal chalcogenides, metals, and perovskites.^23,38,39^ Although simple to perform, each conversion route requires careful design of the reaction conditions to ensure that full chemical conversion is achieved with complete preservation of the 3D shape and fine details.

One of the compound classes that would be highly desirable for accessing via such a conversion route is metal selenides. Metal selenides are of great interest because the small bandgaps of these semiconductors make them ideal for (photo-)catalytic, optical, and photovoltaic applications.^40−45^ Control over the 3D morphology is highly desirable to optimize and enhance these functionalities, such as light trapping for enhanced photoactivity and tunable transport for catalytic surfaces.^46^ However, despite tremendous progress,^42,44,47,48^ controlling and tuning of the 3D shape of metal selenides remains challenging.

Based on this motivation, we here present a proof of concept for a self-organization/conversion strategy to produce 3D shape-controlled architected metal selenides. To achieve successful chemical conversion, we design a sequential series of shape-preserving ion-exchange reactions to transform the nanocrystals in self-organized architected BaCO_3_/SiO_2_ nanocomposites into cadmium selenide. Complete ion exchange with shape preservation—while avoiding non-reactivity, Ostwald ripening, dissolution, and recrystallization—requires minimal distortion of the crystal structure and shifting the equilibria to rapidly drive the reaction toward the desired product.^23,49^ Based on these considerations, we propose a two-step ion-exchange reaction (Figure 1B): (i) cation exchange from BaCO_3_ to CdCO_3_ and (ii) anion exchange from CdCO_3_ to (hexagonal) CdSe. The exchange from BaCO_3_ toward CdCO_3_ is already known^23^ and yields minimal deformation of the crystal lattice during the change from orthorhombic BaCO_3_ to trigonal CdCO_3_ (Figure 1C), while the nanocomposite layout offers both mechanical stability and chemical reactivity. For the exchange from CdCO_3_ toward CdSe, there is no reaction known. However, we realize that the ionic nature of selenium^40,50^ and the removal of CO_2_ suggest that this conversion is possible via CdCO_3_(s) + 2Se(g) → CdSe(s) + SeO(g) + CO_2_(g). Following Le Chatelier’s principle, this reaction equilibrium is shifted toward the desired CdSe by removing the gaseous SeO and CO_2_ products from the reaction mixture. Moreover, comparison of the crystal structures suggests that minimal changes of the crystal lattice are sufficient to enable the reordering from trigonal CdCO_3_ to hexagonal CdSe (Figure 1C).

## Results and Discussion

**Figure 2:**
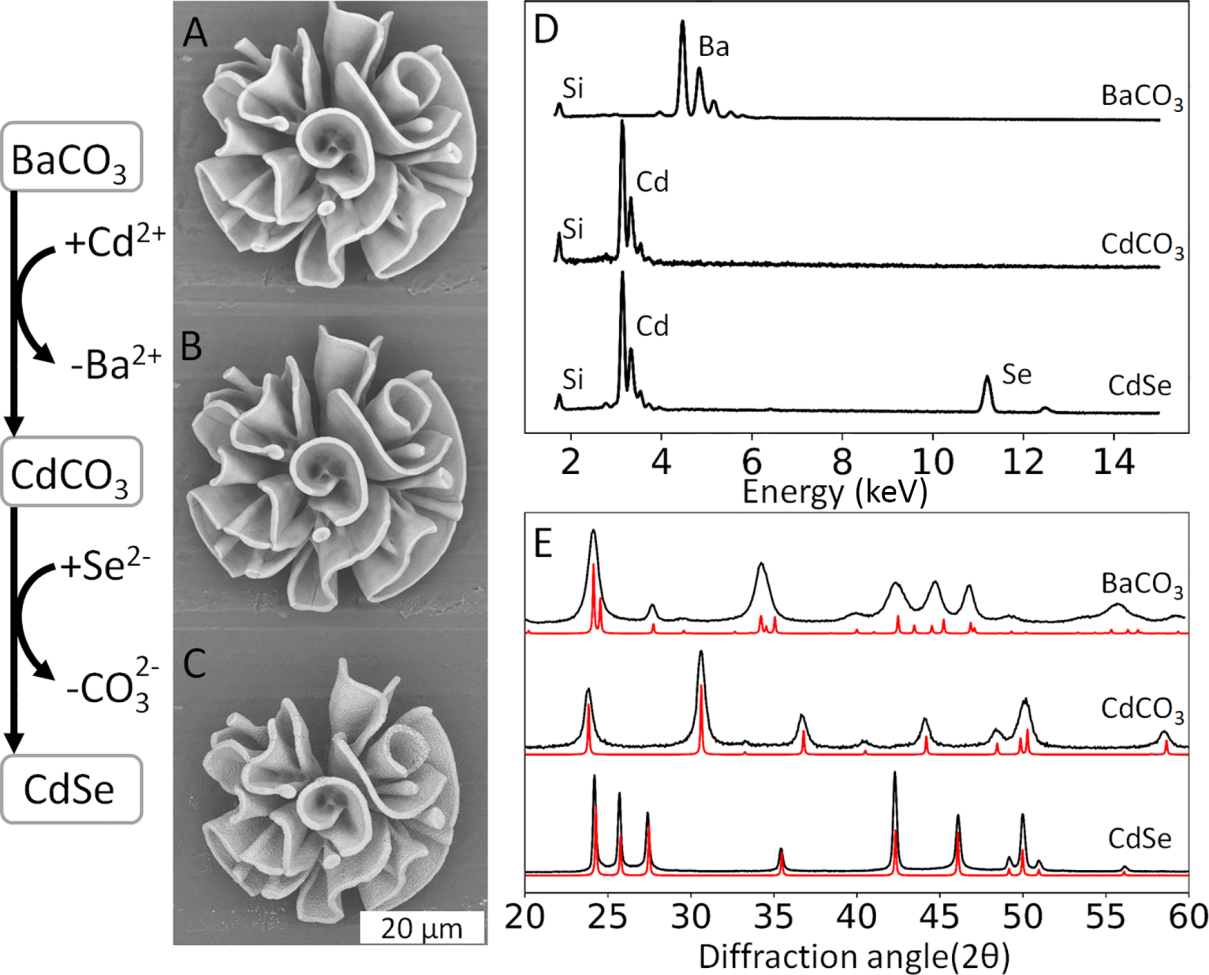
Shape-preserving conversion from BaCO_3_ to CdSe. (A) SEM backscatter micrographs of the same coral-shaped nanocomposite showing shape preservation and volume adjustment after each reaction step from BaCO_3_/SiO_2_ to (B) CdCO_3_/SiO_2_ and (C) CdSe/SiO_2_. (D) EDS analysis and (E) XRPD analysis with the red lines showing the reference peaks indicate complete chemical conversion during each reaction step from orthorhombic BaCO_3_ to trigonal CdCO_3_ and hexagonal CdSe. Broadening and coalescence of the BaCO_3_ diffraction peaks at 19, 24, and 34° likely result from absorption of silica into BaCO_3_.^51^

We demonstrate the proof of principle of shape-preserving conversion reactions toward metal selenides starting from BaCO_3_/SiO_2_ coral-shaped nanocomposites. These nanocomposites spontaneously self-assemble on an aluminum substrate placed in a solution of barium chloride dihydrate (BaCl_2_·2H_2_O, 74 mg, 20 mM) and sodium metasilicate (Na_2_SiO_3_, 16 mg, 8.7 mM) in water (15 mL).^23,49^ We allow CO_2_ from the atmosphere to diffuse into the solution, which onsets the coprecipitation of BaCO_3_ and SiO_2_ (Figure 1A). After 90 min, the substrate is removed from the solution and washed with water and acetone to isolate the precipitate for further analysis. As expected, X-ray powder diffraction (XRPD) confirms the precipitation of BaCO_3_ in the wurtzite crystal structure, while scanning electron microscopy (SEM) and energy-dispersive X-ray spectroscopy (EDS), respectively, show well-defined coral-like forms consisting of BaCO_3_ and SiO_2_ in an approximately 4:1 atomic ratio (Figure 2D,E, see Supporting Information).

Based on our sequential exchange route (Figure 1B), in the first conversion step, we exchange barium for cadmium to yield cadmium carbonate (CdCO_3_). Following previously developed methods,^23^ we perform this cation exchange by immersing the aluminum substrate with BaCO_3_/SiO_2_ coral-like forms into an aqueous solution with cadmium ions (5 mM). This reaction is driven toward the formation of CdCO_3_ because of the 1000-fold lower solubility of CdCO_3_ (*K*_sp, CdCO_3__ = 1.0 × 10^–12^) compared to BaCO_3_ (*K*_sp, BaCO_3__ = 2.58 × 10^–9^), the high concentration of Cd^2+^ ions in the solution, and the similarities in the anionic framework between the orthorhombic BaCO_3_ with HCP packed carbonate and trigonal CdCO_3_ with CCP packed carbonate. The reaction is stopped after 12 min by washing the substrate with water and acetone. EDS and XRPD analyses of the resulting structures confirm virtually complete conversion of BaCO_3_ to CdCO_3_ (Figure 2) (see Supporting Information Section S4 for more details), while comparison of SEM backscatter micrographs of the same architecture before and after conversion shows excellent preservation of the initial coral-like form.

Following our scheme (Figure 1B), in the second step, we exchange the carbonate group for selenium. To this aim, we position the CdCO_3_ nanocomposites in a chemical vapor deposition (CVD) tube furnace with 99.99% pure selenium powder. To avoid undesired side reactions, oxygen is removed from the reaction vessel by reducing the pressure (<1 mbar) followed by flushing the tube with nitrogen gas (N_2_). Subsequently, the CVD furnace is heated to 500 °C with a heating rate of between 0.5 and 50 °C per minute and at a pressure between 20 and 50 mbar with 6 CC/min N_2_ flow. After 1 h, the furnace is cooled down to room temperature. The initial CdCO_3_/SiO_2_ composites have changed color from white to dark gray/black, which is indicative of the formation of CdSe.^40,52^

To confirm the conversion toward CdSe, the nanocomposites are analyzed using EDS, SEM, and XRPD (Figure 2). EDS analysis shows an approximately 1:1 ratio between cadmium and selenium, hence indicating full conversion toward CdSe. Furthermore, the approximate 4:1 atomic ratio between metal and silica of the original BaCO_3_/SiO_2_ composite is maintained, indicating that no significant amount of cadmium is lost from the composite by accidental sublimation during the heating of selenium conversion (see Supporting Information Section S6 for details). XRPD analysis confirms complete conversion to hexagonal CdSe (Figure 2).

Based on the XRPD pattern, the crystal domain size is calculated before and after conversion using the Scherrer equation (see Supporting Information Section S11 for details). For the conversion from BaCO_3_ to CdCO_3_, we find that the crystal domain size increases slightly from 18 to 21 nm. For the conversion from CdCO_3_ to CdSe, we find that the crystal domain size of the resulting CdSe depends on the reaction pressure. For a pressure of 20 mbar, we find that only minor growth of the crystal domain size occurs (∼2 nm for starting sizes of 20–30 nm), while for higher pressures (50 mbar), a significant increase in the crystal domain size is observed (∼10 nm) (see Supporting Information Sections S3 and S11 for more details). Comparison of SEM backscatter micrographs of the same architecture before and after the conversion shows that the original shape and fine details are well-preserved but shrunken. We quantify this volume change using previously developed methods^49^ and find that the volume of the composite is shrunken by approximately 25%, which is slightly more than the expected 20% volume change from the crystal lattice volume (see Supporting Information Section S13 for more details).^49^ Consistent with previous observations for metal chalcogenides and perovskites,^23,25,38,39,49^ the nanocomposite layout thus facilitates shape-preserving conversion toward CdSe, while the microscopic volume adapts to volume changes of the crystal lattice.

To benchmark the semiconductor character of the resulting composites, we determine the bandgap of the coral-shaped CdSe nanocomposites using ultraviolet–visible (UV–vis) spectroscopy in an integrating sphere setup (see Supporting Information Section S10 for details). Depending on the reaction conditions, we find a bandgap between 1.64 eV for slow heating rates (0.5 °C/min) and 1.68 eV for fast heating rates (50 °C/min) (see Supporting Information Sections S10 and S11 for details). These bandgaps are slightly down-shifted compared to the expected bulk bandgap of 1.74 eV. To further investigate the cause of this shift in the bandgap, we perform XRPD and photoluminescence (PL) analyses on these nanocomposites. We observe no significant shifting or broadening of peaks in the XRPD diffractogram, making bulk strain unlikely. In the PL spectrum, we detect luminescence at the expected wavelength of 720 nm for fast heating rates, while we find near-complete quenching of the PL signal for slow heating rates (see Supporting Information Section S12 for more details). Combined, these results suggest that the downshift of the bandgap is likely caused by surface effects (e.g., surface defects and surface strain), which mainly occur during slow conversion of CdCO_3_ to CdSe.^53−56^ Overall, our conversion strategy thus yields shape-controlled composites assembled from hexagonal CdSe with minimal growth of the crystal domain size and a bandgap that is tunable by the reaction conditions.

The here-developed CdSe/SiO_2_ nanocomposites can serve as a starting point toward other shape-controlled metal selenides. To demonstrate this potential, we investigate the archetypical cation exchange from CdSe to silver selenide (Ag_2_Se). This reaction is driven by the similarities in the crystal structures between the hexagonal CdSe and orthorhombic Ag_2_Se and the ability of CdSe to accept silver cations.^57−59^ We find that well-established ion-exchange routes for individual nanocrystals result in incomplete conversion for our nanocomposites, which we contribute to slower transport of ions in and out of the composite. We envisage that the silica matrix of the nanocomposites can support longer and harsher reaction conditions to drive the conversion reactivity to completion while maintaining the initial shape.

**Figure 3:**
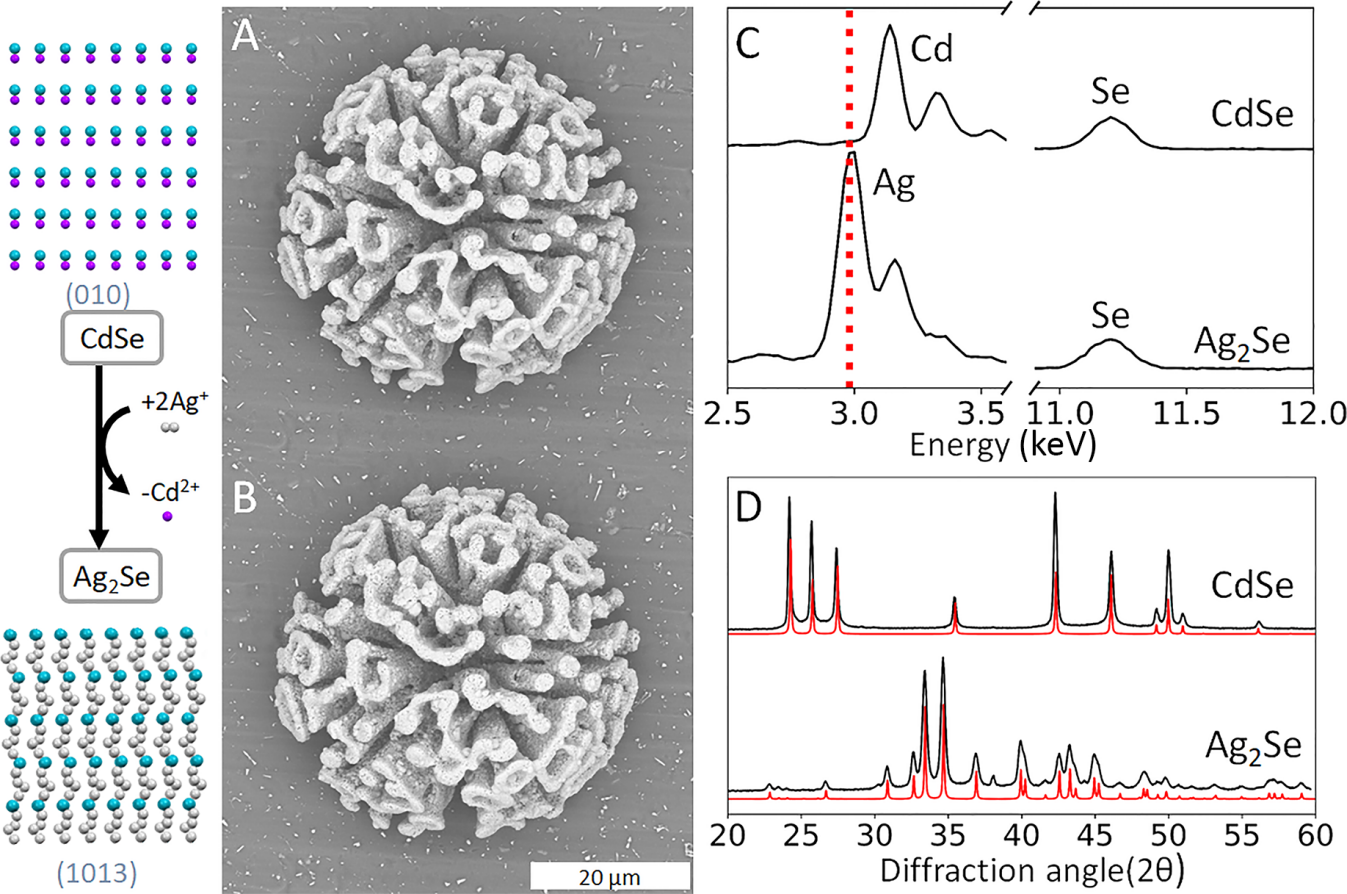
Shape-preserving conversion of CdSe to Ag^2^Se. (A) SEM backscatter micrograph of the same coral-shaped nanocomposite showing shape and volume preservation from CdSe/SiO_2_ to (B) Ag_2_Se/SiO_2_. (C) EDS analysis of both composites. Due to the main X-ray peak of Cd and Ag being close (3.133 vs 2.984 keV), a red line has been drawn to indicate the main peak of Ag. Deconvolution shows near-complete conversion (see Supporting Information Section S7). (D) XRPD analysis with the red lines showing the reference peaks indicates conversion from hexagonal CdSe to orthorhombic Ag_2_Se.

Based on these insights, we perform the conversion by immersing the CdSe composites into a saturated solution of silver nitrate (AgNO_3_, ca. 0.5 M) in methanol at 55 °C for 2 h. The elevated solution temperature increases the AgNO_3_ concentration to enhance the conversion, while methanol facilitates the removal of cadmium ions from the composite.^59^ EDS analysis shows the exchange of cadmium for silver, with an expected ratio of 2:1 for Ag/Se (Figure 3C), while XRPD analysis of the converted composites confirms the formation of orthorhombic Ag_2_Se (Figure 3D). Comparison of the SEM backscatter micrographs shows excellent shape preservation with only a minimal increase of the volume of the composite, which is consistent with the expected minimal (<2%) increase of the crystal lattice volume from hexagonal CdSe to orthorhombic Ag_2_Se.^49^ Our conversion strategy thus enables a cascade of ion-exchange reactions toward a wide range of shape-controlled metal selenide compositions.

**Figure 4:**
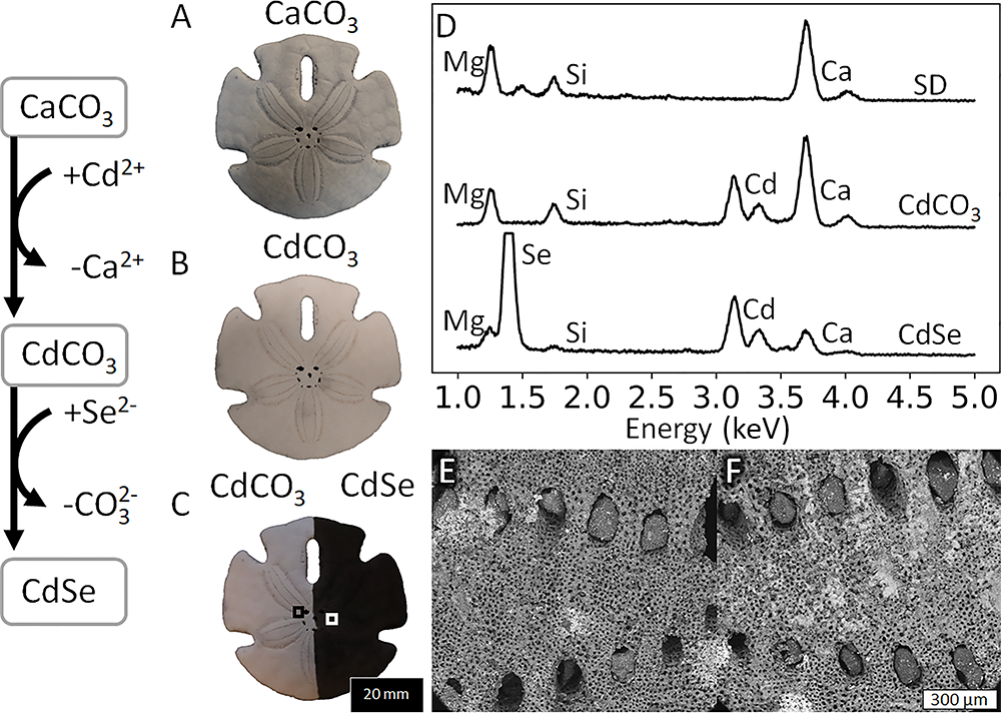
Conversion of a sand dollar to CdSe. Photographs of the same sand dollar (A) before conversion, (B) after ion exchange to CdCO_3_, and (C) after conversion of the right side of the sand dollar into black CdSe. (D) EDS analysis of the original sand dollar (SD) and the subsequent reaction products toward CdCO_3_ and CdSe. (E and F) SEM backscatter micrograph of the selected area on the left and the right, respectively, in (C) showing the preservation of the microstructure after conversion from CdCO_3_ to CdSe.

The universality of our conversion scheme allows us to extend these reactions to naturally occurring biomineral structures made of carbonate salts, many of which have a nanocrystalline layout and composition that are comparable to the artificially synthesized carbonate shapes. To illustrate this concept, we convert a sand dollar biomineral into a CdSe semiconductor via CdCO_3_. The sand dollar consists of magnesium carbonate and calcitic calcium carbonate (MgCO_3_ and CaCO_3_, respectively) and a biological matrix. MgCO_3_ and CaCO_3_ have the same trigonal crystal structure as CdCO_3_, which thus enables minimal distortion of the crystal for cation exchange. Moreover, this reaction is driven to completion because the solubility of the CdCO_3_ (*K*_sp, CdCO_3__ = 1.0 × 10^–12^) is orders of magnitude lower than the original biominerals (*K*_sp, CaCO_3__ = 3.36 × 10^–9^, *K*_kp, MgCO_3__ = 6.82 × 10^–6^). Indeed, immersing the sand dollar in an aqueous solution with cadmium ions (5 mM) for 120 min results in the introduction of Cd according to EDS (Figure 4D). Subsequently, the biomineralized shape is exposed to selenium gas at 500 °C. The color of the sand dollar changes from white to black, which is indicative of the formation of CdSe (Figure 4A–C). EDS analysis shows the presence of Cd and Se in the expected 1:1 ratio, confirming the successful conversion toward CdSe (Figure 4D), while SEM analysis shows the preservation of the porous microscopic structure (Figure 4E,F). These results thus demonstrate that our conversion reactions can readily be applied to mineral architectures of biological origin.

## Conclusions and Outlook

Here, we present a two-step strategy toward metal selenides with controllable 3D shapes. First, we direct the self-organization of BaCO_3_ nanocrystals with a SiO_2_ matrix into nanocomposites with user-defined 3D shapes. Second, we perform a series of ion-exchange reactions to completely convert the BaCO_3_ nanocrystals into CdSe and Ag_2_Se with preservation of the initially programmed 3D shape.

Our conversion strategy for nanocomposite ensembles of nanocrystals has distinct differences compared to the previously developed exchange reaction on individual nanocrystals and superlattices. While individual nanocrystals are directly in contact with the reaction solution, most nanocrystals in composites are embedded in a matrix, which slows down the exchange of ions. Consequently, complete conversion of the nanocomposites requires longer and harsher reaction conditions. Moreover, conversion of individual nanocrystals requires ligands or complexing agents to prevent undesired ripening of the nanocrystals—in particular under harsh reaction conditions.^40,60,61^ In contrast, for the conversion of nanocomposites, each nanocrystal is enveloped by the silica matrix, which inherently restricts undesired ripening, even during prolonged exposure to harsh reaction conditions. This nanostructure may also help to preserve the macroscopic morphology even though the crystal structure undergoes reordering. Hence, the nanocomposite layout facilitates both mechanical stability and chemical reactivity for the preservation of the microscopic 3D shape and nanocrystalline structure.

We foresee that our shape-controlled conversion strategy toward CdSe and Ag_2_Se opens routes to a wide range of other interesting chemical compositions and complex user-defined architectures. Specifically, CdSe and Ag_2_Se have known conversion pathways toward, among others, Cu_2_Se, Cu_2_SnSe_3_, Li_2_Se, MgSe, PbSe, SnSe, and ZnSe,^32,57−59,62^ thus outlining practical routes toward a large library of desirable compositions for which control over the 3D shape is highly desirable. Moreover, we envisage that more refined control over the 3D morphology can be achieved by steering the self-organization process of the initial BaCO_3_/SiO_2_ shape using already-developed light-controlled processes^22^ to sculpt shapes according to exact, user-defined, designs. Finally, our conversion strategy also works on biominerals, thus opening the perspective to exploit the immense catalogue of biomineralized carbonate salt architectures as a starting point, thereby leveraging nature’s exquisite control over morphogenesis for next-generation high-performance materials.

## Methods

### Growth of BaCO_3_/SiO_2_ Nanocomposites

A substrate (e.g., 2 × 2 cm slide of aluminum) was vertically positioned in a 100 mL beaker containing BaCl_2_ dihydrate (74 mg, 0.3 mM) and Na_2_SiO_3_ (16 mg, 0.13 mM) dissolved in 15 mL of water. The reaction vessel was loosely covered with a Petri dish to allow CO_2_ from the air to slowly diffuse into the reaction mixture. Typical growth times ranged between 1.5 and 2.0 h after which the substrate was removed from the solution and washed with deionized water.

### Conversion to CdCO_3_/SiO_2_ Nanocomposites

Cadmium chloride (458 mg, 50 mM) was dissolved in 50 mL of demineralized water. A substrate containing fresh BaCO_3_ nanocomposites was placed in the solution for 12 min. The resulting CdCO_3_ nanocomposites were washed in two demineralized water baths followed by an acetone bath.

### Conversion to CdSe Nanocomposites

CdCO_3_ nanocomposites were placed in a single-zone tube furnace. An alumina boat, loaded with 99.99% pure selenium metal, was added to the single-zone tube furnace. The furnace was purged of oxygen and filled with nitrogen gas until a pressure of 20 mbar was reached. This pressure was maintained with a 6 sccm flow of N_2_, and the furnace was heated to 500 °C at a rate of 50 °C/min and maintained at this temperature for 1 h.

### Conversion to Ag_2_Se Nanocomposites

Silver nitrate (AgNO_3_, 4 g, 0.5 M) was dissolved in 50 mL of methanol at 55 °C and filtered to remove any undissolved AgNO_3_. A substrate containing CdSe nanocomposites was placed in the solution for 120 min. The resulting Ag_2_Se nanocomposites were washed in methanol and allowed to dry by air.

### Scanning Electron Microscopy

The nanocomposites on aluminum substrates were directly loaded into the SEM without pre-treatment. SEM images were obtained using an FEI Verios 460 equipped with an Everhart–Thornley detector, a circular backscatter detector, and an Oxford X-Max^n^ EDS system with Aztec V6.0 software (Oxford Instruments) using the built-in deconvolution algorithms. The images were recorded at 10 kV with 100 pA current. EDS spectra were measured at 30 kV using a 100 pA current.

### X-ray Diffraction Characterization

XRPD characterization was performed using a Bruker D2 Phaser (Bragg–Brentano geometry) using a Kα Cu X-ray source with an emission energy of 8.0415 keV. Diffracted X-rays were detected using a Lynxeye detector and were collected for at least 3 h and up to 48 h with a scan interval (Δ2θ) of 0.02° for full diffractogram measurements or 0.002° for Scherrer analysis. XRPD was measured on a 511 index silicon wafer using a 1 mm knife and a 0.6 mm slit on nanocomposites grown on the meniscus of a growth solution (see Supporting Information Section S2) rather than structures grown on a substrate to maximize the signal-to-noise ratio in the XRPD measurement.

### Photoluminescence Measurement

PL spectra were taken by exciting a sample with a 405 nm Thorlabs S1FC405 diode laser through a WiTEC Alpha300 SR confocal microscope. The laser is focused on the sample with a P5-305A-PCAPC1 optical fiber. PL was measured with a UHTC 300VIS WiTEC spectrometer.

## Supporting Information Available

Growth of BaCO_3_/SiO_2_ nanocomposites; SEM of BaCO_3_/SiO_2_ nanocomposites; scaled-up growth of BaCO_3_/SiO_2_ nanocomposites; conversion to CdCO_3_ nanocomposites; determining the barium content in CdCO_3_ nanocomposites; conversion to CdO nanocomposites; conversion to CdSe nanocomposites; conversion to Ag_2_Se nanocomposites; EDS line scan of Ag, Cd, and Se; bandgap determination; Scherrer equation; investigation of the bandgap shift; calculating the expected microscopic volume change; influence of reaction conditions on the grain domain size and the bandgap; and EDS, XRPD, UV–vis, and PL spectra (PDF)

## Abbreviations

XRPD: X-ray powder diffraction
EDS: energy-dispersive X-ray spectroscopy
SEM: scanning electron microscopy
IR: infrared spectroscopy
SD: sand dollar
PL: photoluminescence
