# Supplementary material for: Architected Metal Selenides via Sequential Cation and Anion Exchange on Self-Organizing Nanocomposites
Source: Chem Mater. 2023 Mar 8;35(6):2394–401. doi: 10.1021/acs.chemmater.2c03525 (PMC10061662; doi:10.1021/acs.chemmater.2c03525)
Supplement: Supplementary file 1 — cm2c03525_si_001.pdf [file cm2c03525_si_001.pdf]

Supporting Information

Architected metal selenides via sequential cat- and anion exchange on self-organizing nanocomposites

Arno van der Weijden[1], Anne-Sophie Léonard[1], Willem L. Noorduin[1,2]*

1: *AMOLF, Science Park 104, Amsterdam 1098 XG, The Netherlands*

2: *Van 't Hoff Institute for Molecular Sciences, University of Amsterdam, Science Park 904, Amsterdam 1090 GD, The Netherlands*

* corresponding author: w.noorduin@amolf.nl

S1. Growth of $BaCO_3/SiO_2$ nanocomposites

$BaCO_3/SiO_2$ nanocomposites on a substrate were prepared as described in previous literature.[1] In short, a substrate (e.g., 2x2 cm slide of aluminium) was vertically positioned in a 100 mL beaker containing $BaCl_2$ dihydrate (74 mg, 0.3 mM) and Na_2SiO_3 (16 mg, 0.13 mM) dissolved in 15 mL of water. The reaction vessel was loosely covered with a Petri dish to allow CO_2 from the air to slowly diffuse into the reaction mixture. Typical growth times ranged between 1.5-2.0 hours after which the substrate was removed from the solution and washed with deionized water. The resulting $BaCO_3/SiO_2$ nanocomposites are then instantly converted in the next step (Figure S1).

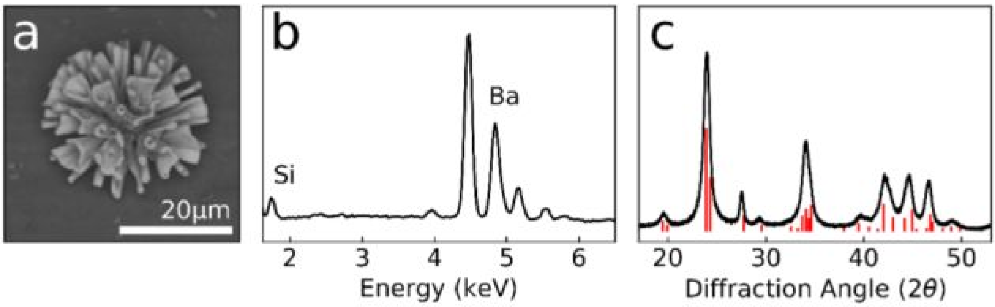

Figure S1: $BaCO_3$ nanocomposites. (A) SEM, (B) EDS, (C) XRD with reference lines depicted (COD 1000033). Reprinted with permission from Hendrikse, H. C.; van der Weijden, A.; Ronda-Lloret, M.; Yang, T.; Bliem, R.; Shiju, N. R.; van Hecke, M.; Li, L.; Noorduin, W. L. Shape-Preserving Chemical Conversion of Architected Nanocomposites. *Adv. Mater.* **2020**, *32* (52), 1–7. Copyright 2020 John Wiley and Sons.

S2. Scaled up growth of $BaCO_3/SiO_2$ nanocomposites

A solution of $BaCl_2$ (7.4 g, 30 mM) in 300 mL of water was added to a solution of Na_2SiO_3 (1.6 g, 13 mM) in 1200 mL of water. This solution was shortly stirred and poured in a metal tray (30x50x10 cm) to maximize the surface area in contact with air, while keeping at least 1 cm of depth to the solution. The solution was left for 1.5 hours with the tray covered polycarbonate lid which is perforated with 0.3 mm holes. The resulting nanocomposites floating on the meniscus were separated from the solution via vacuum filtration. For further exchange reactions, the microstructures were instantly removed from the filtration paper with a spatula and transferred directly into the exchange solution (Figure S2).

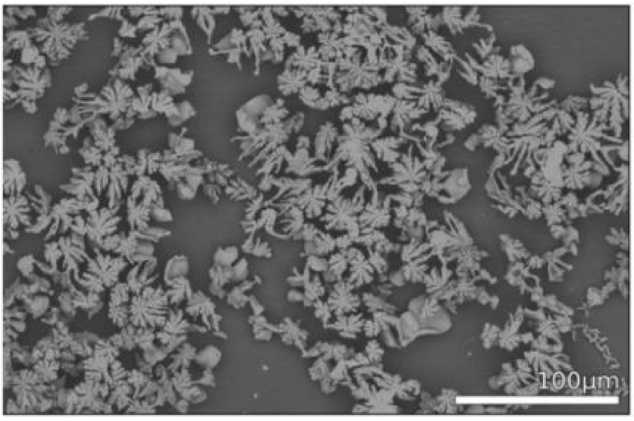

Figure S2: SEM of $BaCO_3/SiO_2$ nanocomposites grown on the meniscus. Reprinted with permission from Hendrikse, H. C.; van der Weijden, A.; Ronda-Lloret, M.; Yang, T.; Bliem, R.; Shiju, N. R.; van Hecke, M.; Li, L.; Noorduin, W. L. Shape-Preserving Chemical Conversion of Architected Nanocomposites. *Adv. Mater.* **2020**, *32* (52), 1–7. Copyright 2020 John Wiley and Sons.

S3 Conversion to $CdCO_3$ nanocomposites

Cadmium chloride (458 mg, 50 mM) was dissolved in 50 mL demineralized water. A substrate containing fresh $BaCO_3$ nanocomposites was placed in the solution for 12 minutes. The resulting $CdCO_3$ nanocomposites were washed in two demineralized water baths followed by an acetone bath. The structures were analyzed using SEM, EDS and XRD (Figure S3). EDS analysis showed a Cd:Si ratio of 75.8:24.2 and XRD Scherrer analysis reveals a slight increase in crystal domain size as compared to the $BaCO_3$ from 18 nm to 21 nm.

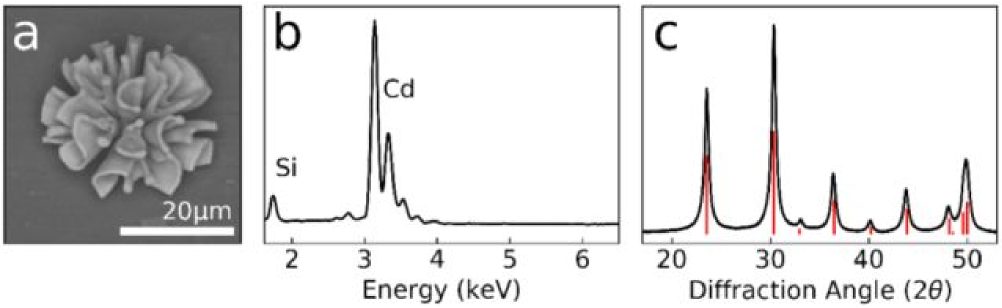

Figure S3: $CdCO_3$ nanocomposites. (A) SEM, (B) EDS, (C) XRD with reference lines depicted (AMCSD 0004324). Reprinted with permission from Hendrikse, H. C.; van der Weijden, A.; Ronda-Lloret, M.; Yang, T.; Bliem, R.; Shiju, N. R.; van Hecke, M.; Li, L.; Noorduin, W. L. Shape-Preserving Chemical Conversion of Architected Nanocomposites. *Adv. Mater.* **2020**, *32* (52), 1–7. Copyright 2020 John Wiley and Sons.

S4 Determining the barium content in $CdCO_3$ nanocomposites

$CdCO_3$ nanocomposites were placed in a single zone tube furnace. The pressure in the furnace was reduced to <1 mbar. The temperature was raised to 500°C for 12 hours after which the oven was allowed to cool. The resulting nanocomposites were deprived of cadmium due to the sublimation of CdO. The composition of the structures was analyzed using EDS and they were found to consist of 98-99% of silica with 1-2% barium. Using this ratio, we can calculate that the original barium content was approximately 0.2-0.4%.

S5 Conversion to CdO nanocomposites

$CdCO_3$ nanocomposites were placed in a single zone tube furnace. The furnace was purged of oxygen and filled with nitrogen gas till a pressure of 500 mbar was reached. This pressure was maintained with a 50 sccm flow of N_2 as the furnace was heated to 290°C for 4 hours. The resulting nanocomposites were characterized by SEM, EDS and XRD (Figure S4).

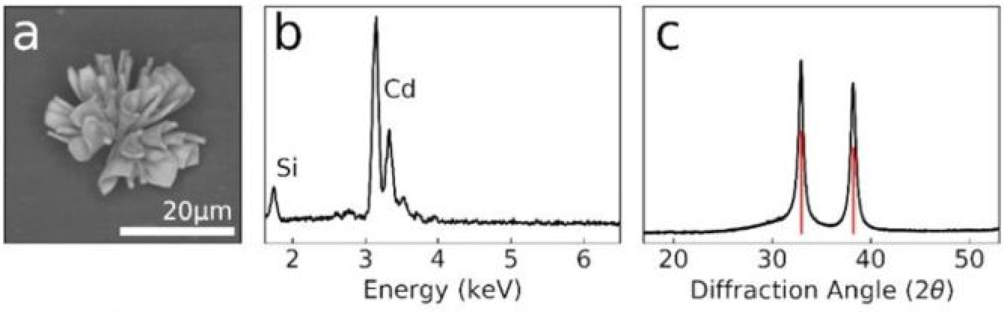

Figure S4: CdO nanocomposites. (A) SEM, (B) EDS, (C) XRD with reference lines depicted (COD 1011003). Reprinted in part with permission from Hendrikse, H. C.; van der Weijden, A.; Ronda-Lloret, M.; Yang, T.; Bliem, R.; Shiju, N. R.; van Hecke, M.; Li, L.; Noorduin, W. L. Shape-Preserving Chemical Conversion of Architected Nanocomposites. *Adv. Mater.* **2020**, *32* (52), 1–7. Copyright 2020 John Wiley and Sons.

S6 Conversion to CdSe nanocomposites

$CdCO_3$ nanocomposites were placed in a single zone tube furnace. An alumina boat was added to the single zone tube furnace. The furnace was purged of oxygen and filled with nitrogen gas until a pressure between 20 and 50 mbar was reached. This pressure was maintained with a 6 sccm flow of N_2 as the furnace was heated to 500°C at a rate between 0.5 and 50°C/min and maintained at this temperature for 1 to 12 hours. The structures used for the conversion to Ag_2Se were created at 20 mbar with a heating rate of 50°C/min and the temperature was maintained for 1 hour. The resulting nanocomposites were characterized by SEM, EDS, XRD and UV-VIS Diffuse Reflectance Spectroscopy (DRS) (Figure S5). EDS analysis on the structure in 2C and 3A showed an atomic ratio for Se:Cd:Si of 44.3:42.1:12.7 with a 0.9% trace of iron and 47.0:42.2:10.3 with a 0.5% trace of iron respectively.

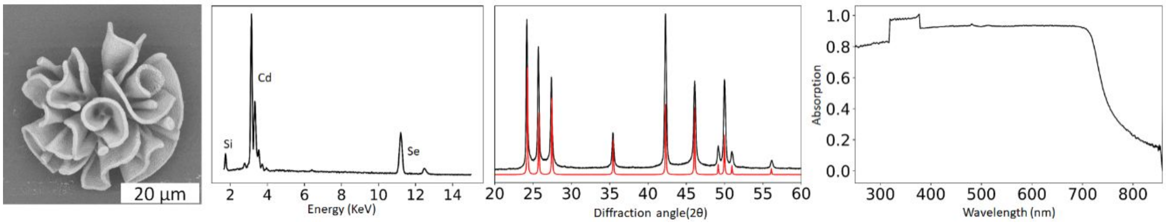

Figure S5: CdSe nanocomposites. (A) SEM, (B) EDS, (C) XRD, with reference lines depicted (COD 9011664) and (D) UV-VIS-DRS for a sample converted at 20mbar with a 50°C/min heating rate.

S7 Conversion to Ag_2Se nanocomposites

Silver nitrate ($AgNO_3$, 4 g, 0.5 M) was dissolved in 50 mL methanol at 55°C and filtered to remove any undissolved $AgNO_3$. A substrate containing CdSe nanocomposites was placed in the solution for 120 minutes. The resulting Ag_2Se nanocomposites were washed in methanol and allowed to dry by air. The structures were analyzed by SEM, EDS, and XRD (Figure S6). Using Oxford Instrument's True-Q® deconvolution algorithm the atomic ratio of elements was determined to be 59%Ag, 33.9%Se, 5.6%Si, and 1.5% Cd with cadmium falling outside the certainty range. An additional deconvoluted linescan was preformed to show the difference in ratio between Ag and Cd (Figure S7)

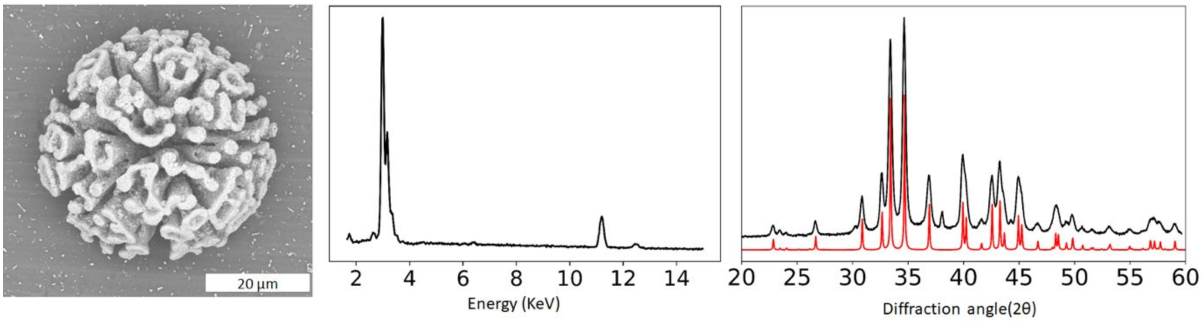

Figure S6: Ag_2Se nanocomposites. (A) SEM, (B) EDS, (C) XRD, with reference lines depicted (COD 2230972)

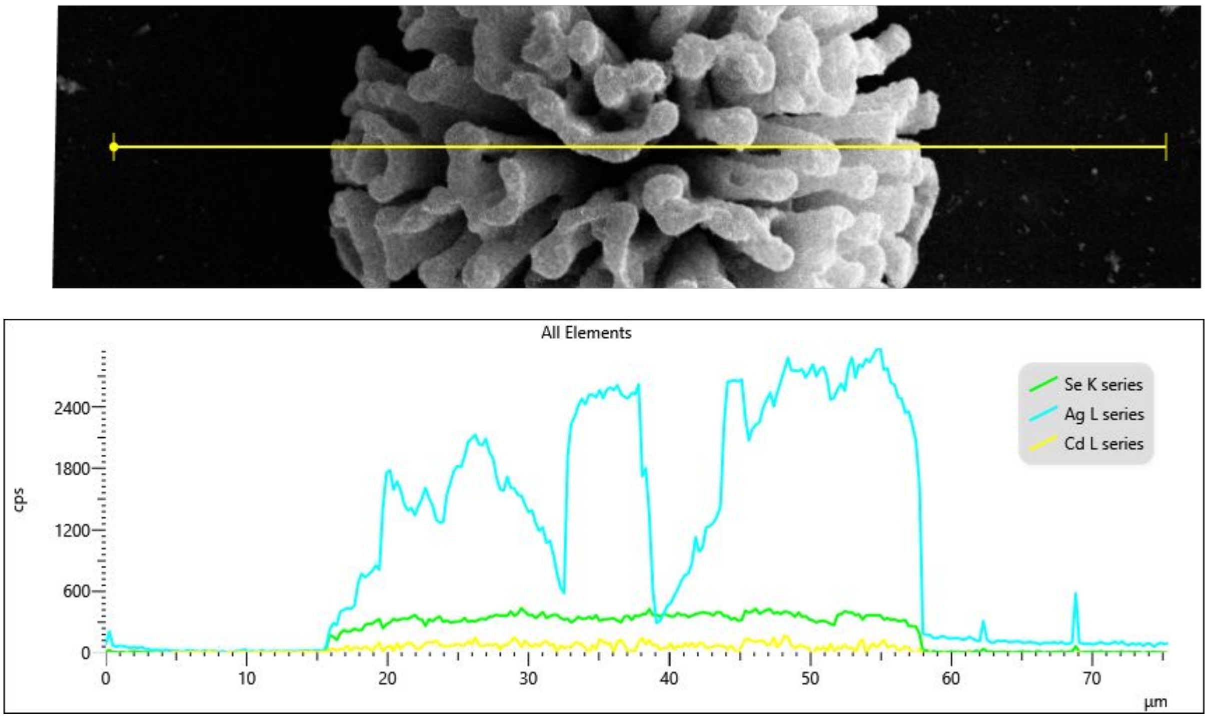

Figure S7: EDS line scan of Ag (L series), Cd (L series) and Se (K series due to overlap with the substrate in the L series). Deconvoluted using Oxford Instrument's True-Q®deconvolution algorithm. By comparing Ag with Cd it becomes clear that the conversion was complete.

The same reaction was attempted using small amounts of acetonitrile in methanol to increase the solubility of $AgNO_3$. Unfortunately, the addition of acetonitrile prevented the formation of orthorhombic Ag_2Se and an amorphous result was obtained. The color for this amorphous material was gray instead of black for the orthorhombic Ag_2Se.

S8 Conversion of bio sample to $CdCO_3$

A sand dollar was bleached using potassium hydroxide (KOH, 1M). It was placed in a solution of $CdCl_2$ (1.4 g, 50 mM) in water (150 mL) for 120 minutes. Afterwards, the sand dollar was placed in a water bath to remove any remaining cadmium ions for 12 hours. Finally, it was dried using an acetone bath. The sample was analyzed by SEM and EDS.

S9 conversion of CdCO3 bio sample to CdSe

The $CdCO_3$ sand dollar was placed in a single zone tube furnace. An alumina boat was added to the single zone tube furnace. The furnace was purged of oxygen and filled with nitrogen gas until a pressure of 20 mbar was reached. This pressure was maintained with a 6 sccm flow of N_2 as the furnace was heated to 500°C at a rate of 50°C/min and maintained at this temperature for 1 hour. The resulting sample was analyzed using SEM and EDS.

S10 Bandgap determination

CdSe powders produced using varied reaction conditions were analyzed using UV-VIS on a PerkinElmer UV/VIS/NIR spectrometer Lambda750 with a 150 mm InGaAs integrating sphere. To calculate the optical bandgap, Tauc plots were constructed. Since the bandgaps were in the visible spectrum, the formula for a direct gap (n=½) was used (Figure S8). The linear fit is defined as:

$$(Ahv)^{\frac{1}{2}} = ahv + b$$

And the corresponding intersection with the horizontal axis, e.g. the bandgap energy, is then defined as:

$$E_{gap} = -\frac{b}{a}$$

The influence of the pressure and heating rate on the bandgap was determined to be dominated by the heating rate with only a minor contribution caused by the pressure and the cooling rate.

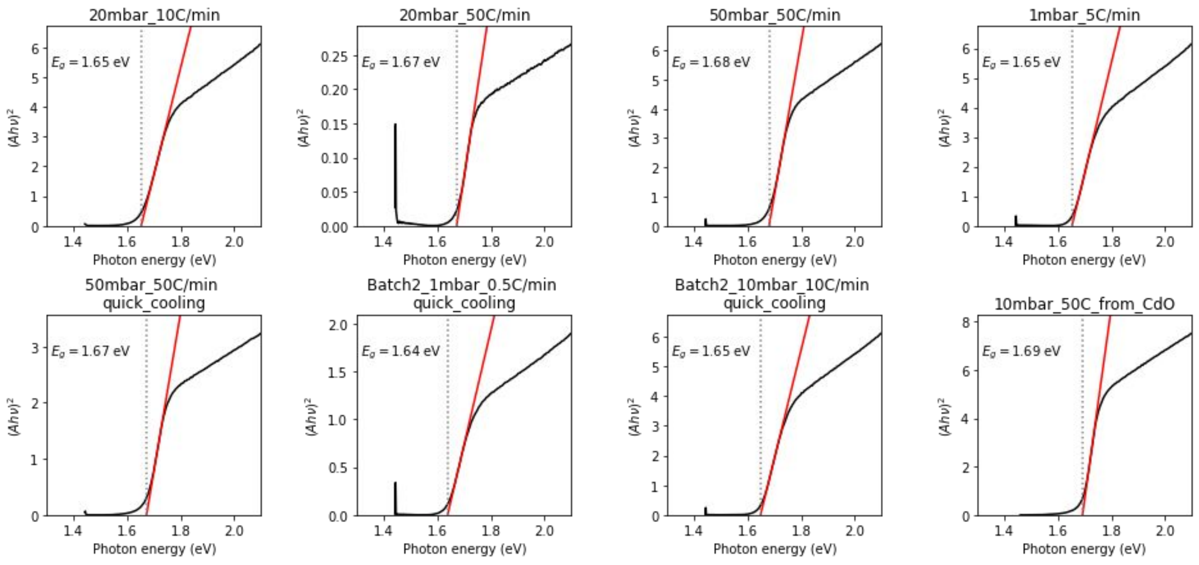

Figure S8: Tauc plots for the direct bandgap with varying conditions.

S11 Scherrer equation

$CdCO_3$ powders were produced in two batches (B1 and B2) and subsequently converted into CdSe under different reaction conditions (Table S1). Using the Scherrer equation the crystal domain size

was calculated from the XRD patterns. This analysis shows that for heating rates above 10 °C/min the crystal domain size was mainly controlled by the pressure.

Sample	Grain domain size	bandgap
Batch 1 $CdCO_3$	29.7 nm	n/a
Batch 2 $CdCO_3$	22.7 nm	n/a
B1, CdSe 1 mbar, 5 °C/min Slow cooling (12h to r.t.)	33.2 nm	1.65 eV
B1, CdSe 20 mbar, 10 °C/min Slow cooling (12h to r.t.)	31.9 nm	1.65 eV
B1, CdSe 20 mbar, 50 °C/min Slow cooling (12h to r.t.)	32.4 nm	1.67 eV
B1, CdSe 50 mbar, 50 °C/min Slow cooling (12h to r.t.)	40.1 nm	1.68 eV
B1, CdSe 50 mbar, 50 °C/min, Quick cooling (1h to r.t.)	40.1 nm	1.67 eV
B2, CdSe 10 mbar, 10 °C/min, Quick cooling (1h to r.t.)	30.5 nm	1.64 eV
B2, CdSe 1 mbar, 0.5 °C/min, Quick cooling (1h to r.t.)	29.7 nm	1.64 eV

Table S1: Influence of reaction conditions on the grain domain size and the bandgap.

S12 investigation of the bandgap shift

To further investigate the cause of the measured bandgap shift based on the heating rate we performed XRD analysis in the range of 2θ = 10-80° for sample batch B1, CdSe 1 mbar, 5 °C/min and sample batch B1, CdSe 20 mbar, 50 °C/min (See Figure S9). We found insignificant XRD peak shifts (See Table S2) ruling out strain and we did not find significant peak broadening at 2θ = 42°. To identify the presence of surface defects we measured photoluminescence (PL) (See Figure S10). PL spectra were taken by exciting a sample with a 405 nm Thorlabs S1FC405 diode laser through a WiTEC Alpha300 SR confocal microscope. The laser is focused on the sample with a P5-305A-PCAPC1 optical fiber. The photoluminescence was measured with a UHTC 300VIS WiTEC spectrometer. We detected a PL signal at 720 nm, consistent with literature, for fast heating CdSe and we observe near complete quenching for the slow heating CdSe.

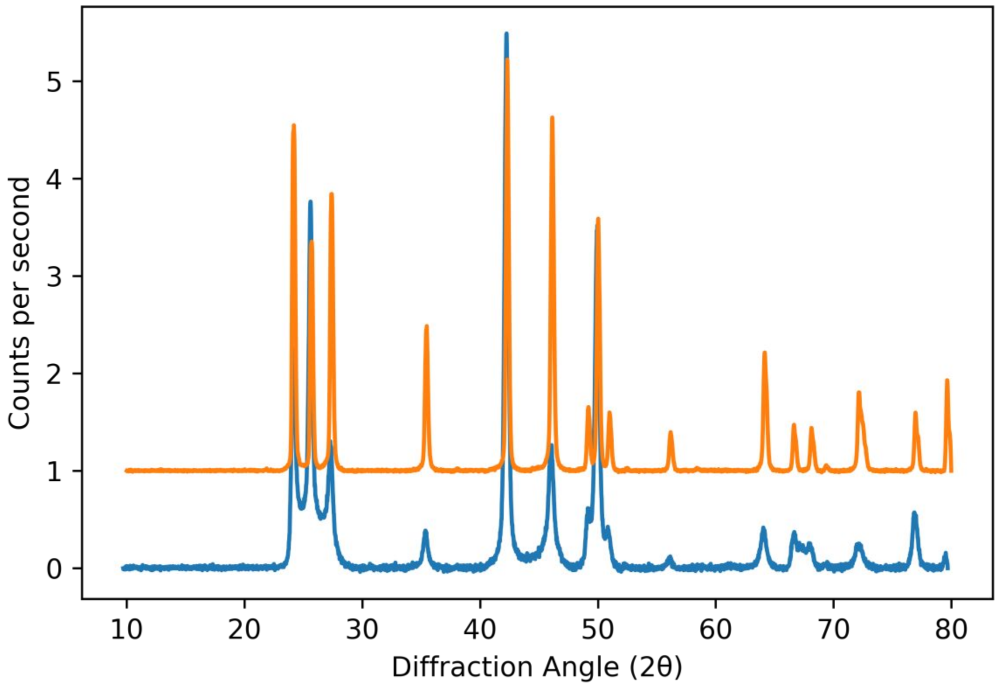

Figure S9: XRD overlay of CdSe converted with a slow heating rate (Blue) and CdSe converted with a fast heating rate (Orange, offset by 1 CPS).

2θ for CdSe formed with fast heating	Δ2θ peak shift between fast and slow heating
24.1948	-0.0026
25.7111	0.0378
27.4094	0.0783
35.4762	0.0985
42.3098	-0.0026
46.1309	-0.0026
49.204	-0.0026
50.0329	0.0176
50.9831	0.0783
56.179	-0.0228
64.165	0.058
66.6518	-0.0835
68.1276	0.1591
72.1509	-0.0228
76.9627	0.0378
79.6517	0.058

Table S2: XRD shift of CdSe formed with a slow heating rate vs CdSe with a fast heating rate, showing minimal peak shifts.

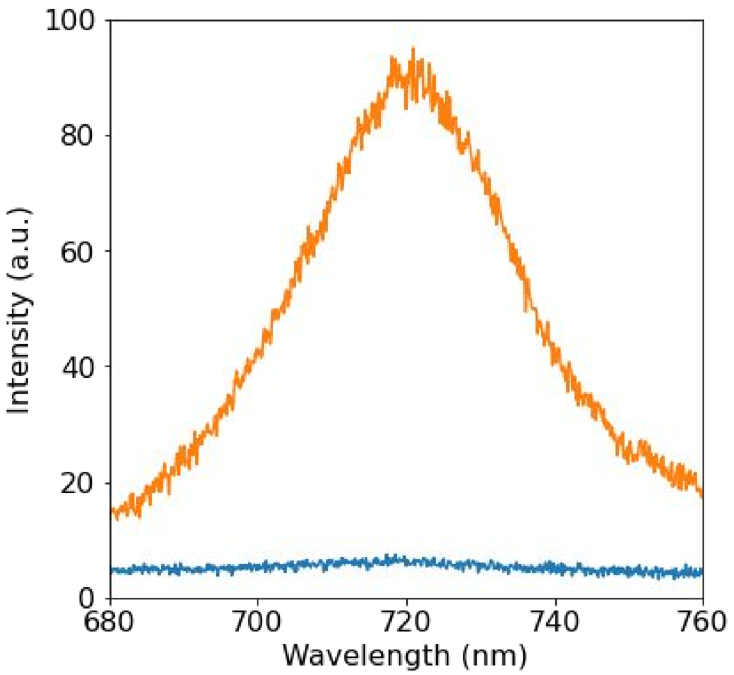

Figure S10: Photoluminescence spectra of CdSe with a slow heating rate (Blue) and CdSe with a fast heating rate (Orange).

S13 Calculating the expected microscopic volume change[1]

The expected volume change of the nanocrystals can be expressed as a change in crystal lattice volume:

$$\theta = \frac{V_{UC}^{F}}{Z^{F}} \Big/ \frac{V_{UC}^{S}}{Z^{S}}$$

with V_{UC}^{S} and V_{UC}^{F} as the unit cell volumes of the starting material and final material, and Z^{S} and Z^{F} as their respective number of formula units per cell.

Part of our structures consists of inert silica. To compensate for this matrix we need to calculate the volume fraction (λ) of the silica matrix. Λ is calculated using the following formula:

$$\lambda = \frac{Vol_{SiO_2}}{Vol_{SiO_2} + Vol_{BaCO_3}}$$

We compute the Vol_{SiO_2} and Vol_{BaCO_3} using the following procedure: First we determine the atomic ratio between the carbonate phase and the silica phase via EDS. This is approximately 0.8:0.2. The atomic ratio is multiplied by the molecular weight and divided by the density. The density of the silica phases from similar sol-gel reactions found in literature is 1.28±0.23 g/cm^3.[2] With this we calculate λ as follows

$$\lambda = \frac{0.2 * 60/(1.28 \pm 0.23)}{0.2 * 60/(1.28 \pm 0.23) + 0.8 * 197.34/4.29} = 0.20 \pm 0.03$$

Finally, we calculate the expected microscopic volume change (ε) as a function of the expected volume change of the nanocrystals following:

$$\varepsilon = (1 - \lambda)\theta + \lambda.$$

References

(1) van der Weijden, A.; van Hecke, M.; Noorduin, W. L. Contraction and Expansion of Nanocomposites during Ion Exchange Reactions. Cryst. Growth Des. 2022, 22 (4), 2289–2293.
